# Longitudinal Viral Load Clustering for People With HIV Using Functional Principal Component Analysis

**DOI:** 10.1155/arat/5890464

**Published:** 2025-01-29

**Authors:** Yunqing Ma, Xueying Yang, Jiayang Xiao, Xiaoming Li, Bankole Olatosi, Jiajia Zhang

**Affiliations:** ^1^Department of Epidemiology and Biostatistics, Arnold School of Public Health, University of South Carolina, Columbia, South Carolina, USA; ^2^South Carolina Smartstate Center for Healthcare Quality, Arnold School of Public Health, University of South Carolina, Columbia, South Carolina, USA; ^3^Department of Health Promotion, Education and Behavior, Arnold School of Public Health, University of South Carolina, Columbia, South Carolina, USA; ^4^Department of Health Services Policy and Management, Arnold School of Public Health, University of South Carolina, Columbia, South Carolina, USA

**Keywords:** FPCA, HIV, longitudinal VL clustering, South Carolina

## Abstract

**Background:** Longitudinal measures of viral load (VL) are critical in monitoring the HIV status. While multiple lab indicators exist for monitoring measures of VL, research on clustering historical/longitudinal VL measures is limited. Analyzing longitudinal VL patterns rather than aggregated measures offers deeper insights into HIV status. This study uses functional data clustering to classify longitudinal VL patterns and characterize each cluster by demographics, comorbidities, social behaviors, and CD4 count.

**Methods:** Adult PWH diagnosed from 2005 to 2015 in South Carolina with a 5-year minimum follow-up were included. We compared functional principal component analysis (FPCA), K-means, hierarchical clustering, and Gaussian mixture models for classification and found FPCA yielded the best results. ANOVA was used to compare VL characteristics, demographics, comorbidities, substance uses, and longitudinal CD4 count across clusters.

**Results:** Results obtained from FPCA could best distinguish the characteristics and patterns into four clusters. A total of 5916 PWH were grouped into long-term VS group (Cluster 1, 17.3%), short-term VS group (Cluster 2, 29.8%), suboptimal VS group (Cluster 3, 28.3%), and viral failure group (Cluster 4, 24.9%). In the long-term VS group with an average of 11-year follow-up, PWH displayed sustained VS (95.3%) and lower mean CD4 count (95.3%) than other clusters. The short-term VS group had shorter follow-up (6 years), more comorbidities (31.4%), and lower percentage of time with low CD4 count (79.9%). In suboptimal VS group, PWH were mostly under 30 years old (44.8%) and Black (77.2%), with relatively lower mean VL (92.9%) and lower VR history (18.4%). In the viral failure group, PWH had higher mean VL (40.6%) and lower mean CD4 count (34.7%).

**Discussion:** The findings highlight the impact of continuous clustering in understanding the distinct viral profiles of PWH and emphasize the importance of tailored treatment and insights to target interventions for all PWH.

## 1. Introduction

Viral suppression (VS), generally defined as maintaining HIV viral load (VL) at undetectable levels [1, 2], is a primary goal of HIV treatment management. The longitudinal measure of VL is important for assessing disease progression, treatment effectiveness, and transmission risk [3, 4]. Maintaining low VL levels can reduce transmission risks and improve health outcomes [5, 6]. Fluctuations in VL can signal changes in disease progression or the effectiveness of antiretroviral therapy (ART), enabling timely adjustments to treatment regimens [7]. Therefore, the potential to proactive monitoring of longitudinal VL measures can serve as a robust metric for evaluating the impact of various interventions., e.g., treatment switch, and becomes an indispensable tool in both clinical care and epidemiological studies that inform public health strategies for HIV control and prevention [8, 9].

Clustering people with HIV (PWH) based on their longitudinal VL offers several significant benefits in the context of HIV treatment [10–12]. First, such clustering enables the identification of distinct trajectories of disease progression and treatment response [11, 13, 14], providing valuable insights into heterogeneity within the patient population. This stratification allows for more personalized and effective treatment regimens, as different clusters may respond differently to ART or other treatment interventions. Second, clustering based on VL can reveal hidden subgroups that are at a higher risk of treatment failure or disease progression [15, 16], thereby facilitating early intervention strategies for these vulnerable populations. Third, it could support efficient resource allocation by helping public health agencies direct interventions and support more effectively when they know which clusters require the most urgent attention [11, 13, 16].

In previous research, researchers performed HIV population case studies using differing schema to classify VL patterns consistent with epidemiological principles, such as the difference in baseline clinical test results (CD4 or VL) among three cohorts, rather than statistical methodologies [17, 18]. Some groups defined clusters based on sustained high VL (SHVL) or durably suppressed VL (DSVL) before comparing patterns of different clusters [19]. However, the clusters may be preconceived because they are based on predetermined criteria that may not account for the complexity and variability of VL. In recent research, PWH have been clustered into time-varying patterns, like sustained low VL, rebounding VL, or SHVL; however, only aggregated VL measures were used, including relative area of viral exposure or weighted recency reliability [16], max VL [20], recent VL [21], and cumulative VL [22, 23], to generalize the dynamic VL patterns. However, current aggregate and/or temporal indicators cannot present the dynamic patterns well since they represent a summary of VL for a period of time rather than continuous changes. Sher et al. reported that it is unreliable to represent the impact of the VL pattern using only timepoint observations because VL change is a dynamic pattern [24]. In addition, the use of aggregate data may yield correlation coefficients exhibiting considerable bias, known as an ecological fallacy [25], encounter information loss [26], and ignore the temporal scope [27].

Longitudinal VL can better reflect the VL dynamics over a period, although there is a challenge in cluster analysis due to the sparsity and inconsistent patient encounters in electronic health records (EHR) intervals [28]. Wang et al. pointed out that EHR data recorded irregularly and continuously can be treated as functional data and analyzed via the functional data analysis (FDA) [29]. Compared with traditional multivariate statistical methods, FDA can easily account for complex, temporal variation [29], extract essential features of the functional data, and can be applied to functional classification [30]. It has been demonstrated to outperform standard clustering methods when used with longitudinal data [31–33]. The goal of this study was to highlight the value of FPCA clustering in understanding the distinct viral profiles of PWH and emphasize the importance of tailored treatment and insights for targeted interventions. Although South Carolina's (SC's) VS rate has increased [34], the rate of VS did not achieve the national goal. In this study, using SC statewide EHR data with over 15 years of follow-up time, we applied the functional principal component analysis (FPCA) to identify the PWH with a similar VL history. Then we compared the FPCA clustering with other existing methods and highlighted the usage of FPCA in longitudinal clustering. Finally, we explored the differences in demographics, comorbidities, social behaviors, and CD4 count among different clusters which could serve as the target for future treatment interventions.

## 2. Methods

### 2.1. Data Source

SC has been compiling a comprehensive electronic HIV/AIDS reporting system for HIV, since 1986. This dataset is maintained by the South Carolina Department of Health and Environmental Control (SC DHEC) through a confidential system specifically designed for HIV/AIDS cases known as the enhanced HIV/AIDS Reporting System (eHARS) [35]. From January 1^st^, 2004, the CDC mandated the inclusion of CD4 count and VL tests [36]. De-identified EHR data from SC DHEC's eHARS and claims data from various payers were linked by the SC Office of Revenue and Fiscal Affairs (SC RFA). More details about the data sources and how they were linked can be found elsewhere [35, 37]. A total of 5,916 PWH who were (1) aged ≥ 18 years old, (2) diagnosed from January 1st, 2005, to December 31st, 2015, in SC, and (3) with a 5-year minimum follow-up from the first VS (VL ≤ 200 copies/mL) to the last VL test were included. Because each PWH took the VL test at varying times, this resulted in a sparse and irregular longitudinal VL history. The average follow-up time is 3616 days (9.90 years (SD: 1144 days (3.13 years))) with an average number of follow-ups being 21.85 (SD: 11.01). Based on the reporting system, we defined VS as VL ≤ 200 copies/mL. When PWH reached the VS, only VL ≤ 200 copies/mL was recorded, and the real numerical value was unavailable.

### 2.2. Variables

The historical VL and CD4 measures were defined across the follow-up time. We defined four aggregated measures for historical VL, including mean VL, max VL, min VL, recent VL, VL percentiles, proportion of the time with VS, and number of viral rebounds (VRs). VR was defined (1) as VL > 200 copies/mL after two consecutive VS, (2) at least one year of follow-up record after first VS, i.e., VL ≤ 200 copies/mL, and (3) at least 90 days apart from the last VS [6]. All VL measures were then categorized into four groups: ≤ 200, 200 to 10,000 to 100,000, and > 100,000 copies/mL.

The demographic variables included gender, age at HIV diagnosis, race, transmission mode for HIV, and residence (urban vs. rural). The clinical variables included substance use (i.e., alcohol use, tobacco use, and illicit drug use) and number of baseline comorbidities at the HIV diagnosis (i.e., comorbidities included hypothyroidism, hypertension, arthritis, COPD, cardiovascular disease, renal disease, diabetes mellitus, obesity, cerebrovascular disease, dyslipidemia, hepatitis C, and hepatitis B). All the clinical variables were identified using ICD-9/10 codes.

In addition, we defined five aggregated measures for historical CD4 count, including baseline CD4 count, max CD4 count, mean CD4 count, nadir CD4 count, and proportion of the time with low CD4 (< 200 cells/μL). All CD4 count measures were categorized into four groups: < 200 cells/μL, 200 to 350 cells/μL, 350 to 500 cells/μL, and > 500 cells/μL. We also constructed the proportion of the time with low CD4 or VS over the total follow-up days, which was categorized as 0, > 0 and ≤ 25%, > 25% and ≤ 50%, > 50% and ≤ 75%, > 75% and < 100%, and 100%.

### 2.3. Approach

We investigated four different clustering methods and selected the best one according to the visualization with multiple VL indicators. The four clustering methods included FPCA [30, 38], K-means clustering [39], hierarchical clustering (Hclust) [40], and Gaussian mixture models (GMM) clustering [41]. For the function clustering, the FPCA was employed to extract variations in sparse longitudinal VL data, which generated the corresponding eigenvalues to capture the underlying variability of longitudinal VL measures and functional principal component (FPC) scores to express the contribution of each FPC to individual observations. Then, the FPC scores were used for clustering because of their ability to distill complex functional data into a manageable number of dimensions that still retain the essential structure of the data. Through this approach, each individual's functional curve is estimated using local smoothing techniques that take into account the sparsity of the data, thus avoiding reliance on interpolation methods that could introduce bias. The EMCluster algorithm [42] was used for the clustering. FPCA clustering was conducted using *fdapace* R package.

Since other variables could not capture the whole VL as a function, we used all aggregated information of VL, i.e., mean VL, max VL, min VL, last VL, and time range from first VS to last VL, for clustering. K-means is designed to partition a dataset into K distinct clusters, where each individual belongs to the cluster with the nearest mean of the aggregated VL information [39]. The hierarchical average linkage clustering method operates by successively merging or dividing individuals based on similarity measures of the aggregated VL, forming a tree-like structure known as a dendrogram [40]. GMM operates under the assumption that the data are generated from a mixture of several Gaussian distributions [41] and that it separates individuals based on the probability of each individual belonging to each cluster.

To explore the similarities and differences among different clusters and validate the proposed clustering method, after getting the clustering labels through four methods, ANOVA was used to test the difference in VL characteristics, demographics, comorbidities, social behaviors, and historical CD4 count during each cluster's follow-up period.

We used R version 4.3.2 for analysis. A two-sided *p* value of 0.05 was employed to determine statistical significance.

## 3. Results

### 3.1. Basic Features of Longitudinal VL

Among 5916 PWH in this study, majority were 18–30 years old (39.9%), male (74.0%), Black (72.2%), identified as men who have sex with men (MSM) (51.3%), and resided in urban areas (83.0%) (Table 1). For the VL measures, most PWH were viral suppressed over the follow-up time (51.3%) and experienced no VR (82.0%). For the CD4 count measures, most PWH had minimum CD4 count < 200 cells/μL (34.6%), baseline CD4 count > 500 cells/μL (36.1%), maximum CD4 count > 500 cells/μL (81.8%), and mean CD4 count > 500 cells/μL (57.6%). 29.2%, 25.0%, and 8.0% of the population had alcohol, tobacco, and illicit drug use history, while 27.2% had some comorbidity history.

### 3.2. Model Comparison

**Figure 1:**
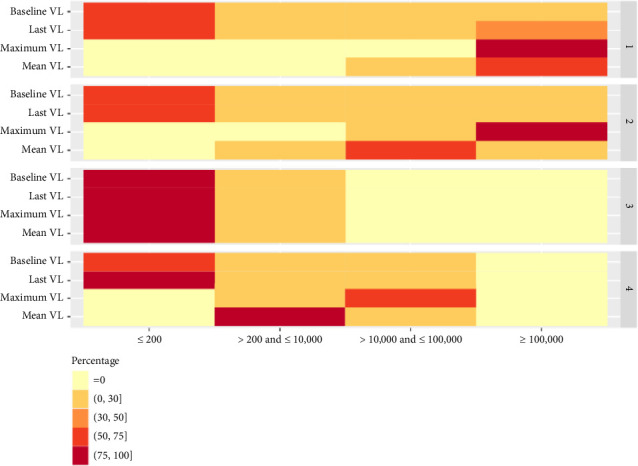
Distribution for VL characteristics (baseline, last, maximum, and mean) using GMM.

**Figure 2:**
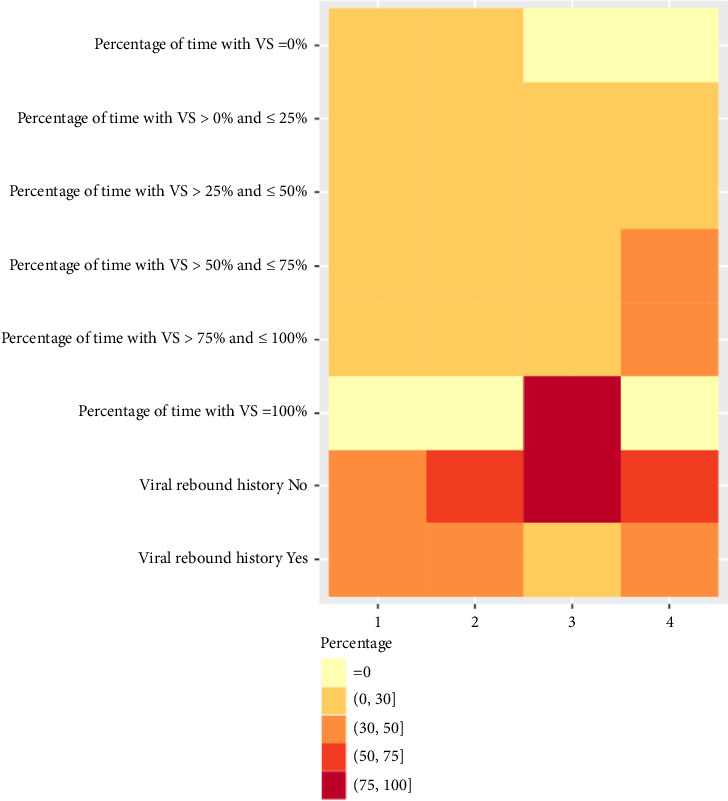
Distribution for VL characteristics (percentage of time with VS and viral rebound history) using GMM.

Using a generalized method of moments (GMM), four distinct clusters were identified with balanced sample sizes of 228 (3.9%), 928 (15.7%), 3650 (61.7%), and 1110 (18.8%), respectively (Figures 1 and 2). From Table 1, differences lied in demographics, comorbidities, social behaviors, and historical CD4 count among four clusters. However, demographics failed to be classified using GMM (Supporting Table 2), since there was no obvious difference between different clusters, i.e., only the third cluster can be distinguished.

For the first cluster, PWH had higher initial VL (> 100,000 cells/mL, 20.2%), higher mean VL (> 100,000 cells/mL, 72.4%), higher initial CD4 count (> 500 cells/μL, 17.5%), and lower minimum CD4 count (< 200 cells/μL, 85.5%). For the second cluster, mean VL mostly fell between 10,000 and 100,000 cells/mL (71.8%). For the third cluster, PWH tended to have lower initial VL (≤ 200 cells/mL, 96.4%), sustained VS over time (83.2%), and higher mean CD4 count (> 500 cells/μL, 67.7%). For the fourth cluster, mean VL most fell in between 200 and 10,000 cells/mL (95.0%). VL percentiles are shown in Supporting Table 1.

Clusters from K-means and Hclust were not informative due to the strong imbalance: the numbers of PWH in the main cluster for K-means and Hclust were 5754 (97.3%) and 5898 (99.7%), meaning that these two methods failed to classify longitudinal VL in this case.

### 3.3. Results Based on the FPCA Clustering

**Figure 3:**
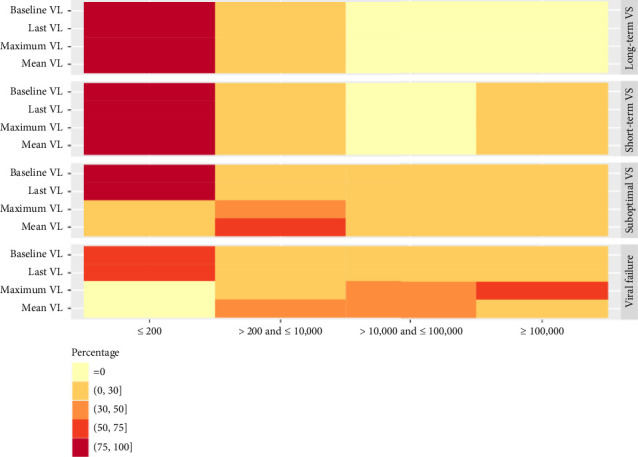
Distribution for VL characteristics (baseline, last, maximum, and mean) using FPCA.

**Figure 4:**
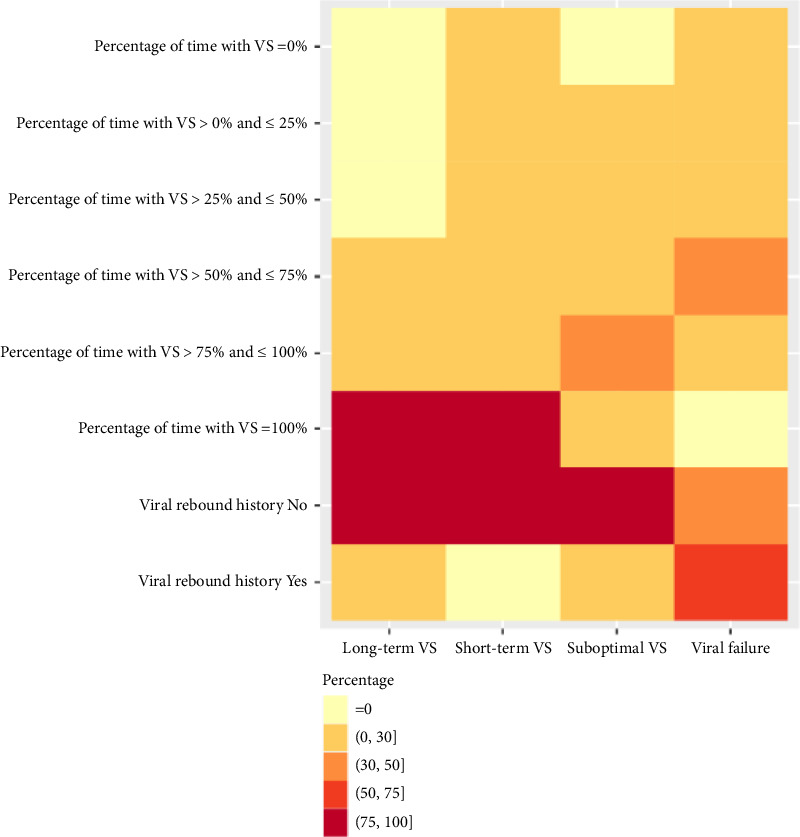
Distribution for VL characteristics (percentage of time with VS and viral rebound history) using FPCA.

**Figure 5:**
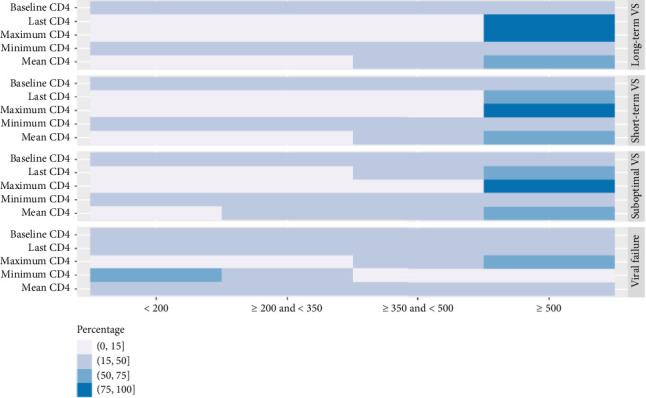
Distribution for CD4 characteristics using FPCA.

A total of 5916 PWH were grouped into four clusters using FPCA clustering method: long-term VS group (Cluster 1, 17.3%), short-term VS group (Cluster 2, 29.8%), suboptimal VS group (Cluster 3, 28.3%), and viral failure group (Cluster 4, 24.9%) (Table 1). In the long-term, VS group with an average of 11-year follow-up, PWH were older (> 50 years old, 17.5%), with lower percentage of Black people compared with other clusters (64.2%), with minimum CD4 count > 500 cells/μL (18.1%) and less alcohol use (20.8%), and were mostly sustained VS (95.3%) (Figures 3 and 4). Characteristics of individuals in the short-term VS group were similar except for shorter follow-up (6 years), a higher percentage of individuals with comorbidity history (31.4%), and higher max CD4 count (> 500 cells/μL, 93.8%) (Figure 5). In the suboptimal VS group, PWH were mostly under 30 years old (44.8%) and Black (77.2%), with relatively lower percentage of individuals with mean VL < 10,000 cells/mL compared with other clusters (92.9%) (Figure 4) and VR history (18.4%). In the viral failure group, demographics were similar to the suboptimal VS group, where majority of PWH had no VS history, lower percentage mean CD4 count > 500 cells/μL (34.7%), and percentage of time with low CD4 count (never had low CD4 count, 41.7%).

## 4. Discussion

The application of FPCA in clustering PWH based on their longitudinal VL patterns has proven to be effective in this study, while other clustering methods (K-means, Hclust, and GMM) failed to distinguish different clusters. The results successfully categorized patients into four distinct clusters with different characteristics: long-term VS group, short-term VS group, suboptimal VS group, and viral failure group. This method has allowed for a more comprehensive understanding of the heterogeneity within the patient population, providing valuable insights into disease progression and treatment responses.

The advantage of FPCA, compared to other clustering methods based only on aggregated VL and other longitudinal measures, lies in its ability to capture the continuity and dynamics of VL, handle sparsity and irregularity in longitudinal data, and accommodate variability and nonlinearity [38, 43]. Namely, FPCA is the only method to cluster functional curves. Dealing with sparse and irregular longitudinal data is a common challenge in epidemiological studies involving EHR data, particularly when analyzing disease trajectories. Continuous clustering via FPCA offers multiple benefits for understanding viral profiles among PWH. It identifies unique VL trajectory patterns, enabling the stratification of PWH into distinct groups that may reflect different infection stages, treatment responses, or disease progressions. These clusters can correlate with varying risk profiles for comorbidity development or virus transmission, informing targeted public health strategies. Furthermore, continuous clustering aids in the predictive modeling of VL changes, guiding clinical decision making and trial designs. It also informs tailored treatment approaches by understanding how different PWH groups respond to treatment and evolve in VL trajectories, ultimately enhancing patient outcomes.

A good clustering method after extracting FPC scores is also important. Therefore, we also compared the use of EMCluster on FPC scores with K-means clustering on the same scores (Supporting Table 3). While the clusters generated by K-means were less balanced than those from EMCluster (with 74.0% of the data in one cluster), they still performed much better than applying K-means solely on aggregated VL data (which resulted in 97.3% in one cluster).

The clustering results obtained in this study are not only statistically meaningful but also clinically interpretable, which is essential in ensuring the relevance and applicability of the findings. The identified clusters align with what has been observed in clinical practice and other research studies [19, 44–47]. For example, PWH in suboptimal VS group may have very similar VL patterns as intermittent and sustained low-level HIV VR [45]; PWH in viral failure group tend to have similar clinical indicators as PWH with SHVL [19]. Thus, the distinction between four groups is consistent with the known variability in treatment responses among PWH.

While continuous clustering using FPCA is a powerful tool for VL pattern clustering, it is important to acknowledge its limitations. Disease and other biological indicators can affect VL dynamics. To achieve a more comprehensive clustering results and clinical understanding, future studies should consider incorporating these confounders, such as longitudinal CD4 counts and comorbidities, into the clustering analysis to account for their influence on VL trajectories. Meanwhile, the study uses a 200 copies/mL threshold as the lower boundary for VL measurements. This threshold may affect the interpretation of VL data and could be a subject of consideration in future research. FPCA can effectively manage sparsity by estimating the underlying functional curves based on the observed data points, without requiring strict regularity in measurements. However, significant irregularity and sparsity continue to affect the effectiveness of the clustering performance [48].

To achieve a more comprehensive clustering results and clinical understanding, future studies should consider incorporating these confounders, such as longitudinal CD4 counts and comorbidities, into the clustering analysis to account for their influence on VL trajectories. Meanwhile, the study uses a 200 copies/mL threshold as the lower boundary for VL measurements. This threshold may affect the interpretation of VL data and could be a subject of consideration in future research.

In conclusion, this study highlights the effectiveness of continuous clustering using FPCA in understanding the distinct viral profiles of PWH. The results obtained provide valuable insights for both clinical practice and public health strategies, emphasizing the importance of tailored treatment and targeted interventions based on individualized disease trajectories.

## Figures and Tables

**Table 1:** Distribution for VL characteristics, demographics, comorbidities, social behaviors, and historical CD4 count for overall and four clusters from FPCA.

Characteristics	Cluster 1	Cluster 2	Cluster 3	Cluster 4	*p* value^1^
	Long-term VS	Short-term VS	Suboptimal VS	Viral failure	
	*n* = 1008 (17.3%)	*n* = 1763 (29.8%)	*n* = 1673 (28.3%)	*n* = 1472 (24.9%)	
Age group (years)					**< 0.001**
≥ 18 and < 30	283 (28.1%)	678 (38.5%)	750 (44.8%)	651 (44.2%)	
≥ 30 and < 40	251 (24.9%)	353 (20.0%)	389 (23.3%)	374 (25.4%)	
≥ 40 and < 50	298 (29.6%)	389 (22.1%)	323 (19.3%)	317 (21.5%)	
≥ 50	176 (17.5%)	343 (19.5%)	211 (12.6%)	130 (8.8%)	
Sex					**< 0.001**
Male	738 (73.2%)	1363 (77.3%)	1249 (74.7%)	1030 (70.0%)	
Female	270 (26.8%)	400 (22.7%)	424 (25.3%)	442 (30.0%)	
Race					**< 0.001**
White	298 (29.6%)	484 (27.5%)	288 (17.2%)	246 (16.7%)	
Black	647 (64.2%)	1173 (66.5%)	1292 (77.2%)	1157 (78.6%)	
Hispanic	48 (4.8%)	67 (3.8%)	63 (3.8%)	47 (3.2%)	
Others	15 (1.5%)	39 (2.2%)	30 (1.8%)	22 (1.5%)	
Risk					**< 0.001**
Heterosexual	257 (25.5%)	301 (17.1%)	380 (22.7%)	406 (27.6%)	
MSM/IDU	48 (4.8%)	78 (4.4%)	100 (6.0%)	104 (7.1%)	
MSM	505 (50.1%)	1000 (56.7%)	849 (50.7%)	683 (46.4%)	
Others	198 (19.6%)	384 (21.8%)	344 (20.6%)	279 (19.0%)	
Region					0.325
Urban	847 (84.0%)	1481 (84.0%)	1376 (82.2%)	1209 (82.1%)	
Rural	161 (16.0%)	282 (16.0%)	297 (17.8%)	263 (17.9%)	
Alcohol use					**< 0.001**
No	798 (79.2%)	1193 (67.7%)	1190 (71.1%)	1008 (68.5%)	
Yes	210 (20.8%)	570 (32.3%)	483 (28.9%)	464 (31.5%)	
Tobacco use					**< 0.001**
No	845 (83.8%)	1265 (71.8%)	1249 (74.7%)	1079 (73.3%)	
Yes	163 (16.2%)	498 (28.2%)	424 (25.3%)	393 (26.7%)	
Illicit drug use					**0.003**
No	949 (94.1%)	1632 (92.6%)	1536 (91.8%)	1327 (90.1%)	
Yes	59 (5.9%)	131 (7.4%)	137 (8.2%)	145 (9.9%)	
Comorbidity history					**< 0.001**
No	761 (75.5%)	1209 (68.6%)	1258 (75.2%)	1075 (73.0%)	
Yes	247 (24.5%)	554 (31.4%)	415 (24.8%)	397 (26.9%)	
Percentage of time with low CD4 count					**< 0.001**
=0%	737 (73.1%)	1383 (79.9%)	1152 (69.7%)	613 (41.7%)	
> 0% and ≤ 25%	241 (23.9%)	260 (15.0%)	397 (24.0%)	483 (32.8%)	
> 25% and ≤ 50%	24 (2.4%)	42 (2.4%)	44 (2.7%)	183 (12.4%)	
> 50% and ≤ 75%	5 (0.5%)	11 (0.6%)	20 (1.2%)	88 (6.0%)	
> 75% and < 100%	0 (0.0%)	11 (0.6%)	9 (0.5%)	39 (2.7%)	
=100%	1 (0.1%)	23 (1.3%)	30 (1.8%)	65 (4.4%)	

*Note:* 2 A two-sided *p* value of 0.05 was employed to determine the statistical significance.

^1^ *p* values were calculated using Pearson's chi-squared test.

## Data Availability

The authors are prohibited from making individual-level data available publicly due to provisions in our data use agreements with state agencies/data providers, institutional policy, and ethical requirements. To facilitate research, we make access to such data available via approved data access requests through the data owners. The data are unavailable externally or for re-release due to prohibitions in data use agreements with our state agencies or other data providers. Institutional policies stipulate that all external requests for data access require collaboration with a USC researcher. For more information or to make a request, please contact Bankole Olatosi, PhD: Olatosi@mailbox.sc.edu. The underlying analytical codes are available from the authors on request.

## Supporting Information

To better show the differences among four clusters from FPCA, we provided the distribution for VL percentiles in Supporting Table 1. Supporting Table 2 shows the distribution for VL characteristics and percentiles, demographics, comorbidities, social behaviors, and historical CD4 count for overall and four clusters from GMM in order to show the advantage of our methods over the GMM clustering. Supporting Table 3 shows the distribution for VL characteristics and percentiles, demographics, comorbidities, social behaviors, and historical CD4 count for overall and four clusters from K-means on the same FPC scores in order to show the advantage of EM Clustering over K-means.
