# Supplementary material for: Longitudinal Viral Load Clustering for People With HIV Using Functional Principal Component Analysis
Source: AIDS Res Treat. 2025 Jan 29;2025:5890464. doi: 10.1155/arat/5890464 (PMC11824709; doi:10.1155/arat/5890464)
Supplement: Supporting Information — Additional supporting information can be found online in the Supporting Information section. [file 5890464.f1.docx]

Supplementary Table 1: Distribution for VL percentiles for four clusters from FPCA

| **Characteristics** | | Cluster 1 | Cluster 2 | Cluster 3 | Cluster 4 | **P value^1^** |
| --- | --- | --- | --- | --- | --- | --- |
|  |  | **Long-term VS** | **Short-term VS** | **Suboptimal VS** | **Viral Failure** |  |
|  |  | n=1,008 (17.3%) | n=1,763 (29.8%) | n=1,673 (28.3%) | n=1,472 (24.9%) |  |
| ***VL 2nd percentile (copies/mL)*** | |  |  |  |  | **<0.001** |
|  | ≤200 | 1,008 (100.0%) | 1,762 (99.9%) | 1,665 (99.5%) | 1,398 (95.0%) |  |
|  | >200 and ≤10,000 | 0 (0.0%) | 1 (0.1%) | 8 (0.5%) | 67 (4.6%) |  |
|  | >10,000 and ≤100,000 | 0 (0.0%) | 0 (0.0%) | 0 (0.0%) | 7 (0.5%) |  |
|  | ≥100,000 | 0 (0.0%) | 0 (0.0%) | 0 (0.0%) | 0 (0.0%) |  |
| ***VL 5th percentile (copies/mL)*** | |  |  |  |  | **<0.001** |
|  | ≤200 | 1,008 (100.0%) | 1,762 (99.9%) | 1,665 (99.5%) | 1,394 (94.7%) |  |
|  | >200 and ≤10,000 | 0 (0.0%) | 0 (0.0%) | 8 (0.5%) | 65 (4.4%) |  |
|  | >10,000 and ≤100,000 | 0 (0.0%) | 1 (0.1%) | 0 (0.0%) | 12 (0.8%) |  |
|  | ≥100,000 | 0 (0.0%) | 0 (0.0%) | 0 (0.0%) | 1 (0.1%) |  |
| ***VL 95th percentile (copies/mL)*** | |  |  |  |  | **<0.001** |
|  | ≤200 | 1,005 (99.7%) | 1,709 (96.9%) | 581 (34.7%) | 29 (2.0%) |  |
|  | >200 and ≤10,000 | 3 (0.3%) | 53 (3.0%) | 786 (47.0%) | 208 (14.1%) |  |
|  | >10,000 and ≤100,000 | 0 (0.0%) | 0 (0.0%) | 257 (15.4%) | 649 (44.1%) |  |
|  | ≥100,000 | 0 (0.0%) | 1 (0.1%) | 49 (2.9%) | 586 (39.8%) |  |
| ***VL 98th percentile (copies/mL)*** | |  |  |  |  | **<0.001** |
|  | ≤200 | 962 (95.4%) | 1,684 (95.5%) | 393 (23.5%) | 0 (0.0%) |  |
|  | >200 and ≤10,000 | 46 (4.6%) | 78 (4.4%) | 780 (46.6%) | 131 (8.9%) |  |
|  | >10,000 and ≤100,000 | 0 (0.0%) | 0 (0.0%) | 393 (23.5%) | 604 (41.0%) |  |
|  | ≥100,000 | 0 (0.0%) | 1 (0.1%) | 107 (6.4%) | 737 (50.1%) |  |
| ^1^ P-values were calculated using Pearson's Chi-squared test. | | | | | | |

Supplementary Table 2: Distribution for VL characteristics and percentiles, demographics, comorbidities, social behaviors, and historical CD4 count for overall and four clusters from GMM

| **Characteristics** | | **Overall**, **N** = 5,916 | Cluster 1 | Cluster 2 | Cluster 3 | Cluster 4 | **P value^12^** |
| --- | --- | --- | --- | --- | --- | --- | --- |
|  |  |  | n=228 (3.9%) | n=928 (15.7%) | n=3,650 (61.7%) | n=1,110 (18.8%) |  |
| ***Age Group (Years)*** | |  |  |  |  |  | **<0.001** |
|  | ≥18 and <30 | 2,362 (39.9%) | 101 (44.3%) | 376 (40.5%) | 1,323 (36.2%) | 562 (50.6%) |  |
|  | ≥30 and <40 | 1,367 (23.1%) | 63 (27.6%) | 260 (28.0%) | 820 (22.5%) | 224 (20.2%) |  |
|  | ≥40 and <50 | 1,327 (22.4%) | 47 (20.6%) | 192 (20.7%) | 867 (23.8%) | 221 (19.9%) |  |
|  | ≥50 | 860 (14.5%) | 17 (7.5%) | 100 (10.8%) | 640 (17.5%) | 103 (9.3%) |  |
| ***Sex*** | |  |  |  |  |  | **0.021** |
|  | Male | 4,380 (74.0%) | 171 (75.0%) | 680 (73.3%) | 2,745 (75.2%) | 784 (70.6%) |  |
|  | Female | 1,536 (26.0%) | 57 (25.0%) | 248 (26.7%) | 905 (24.8%) | 326 (29.4%) |  |
| ***Race*** | |  |  |  |  |  | **/** |
|  | White | 1,316 (22.2%) | 48 (21.1%) | 151 (16.3%) | 940 (25.8%) | 177 (15.9%) |  |
|  | Black | 4,269 (72.2%) | 169 (74.1%) | 731 (78.8%) | 2,489 (68.2%) | 880 (79.3%) |  |
|  | Hispanic | 225 (3.8%) | 9 (3.9%) | 28 (3.0%) | 154 (4.2%) | 34 (3.1%) |  |
|  | Others | 106 (1.8%) | 2 (0.9%) | 18 (1.9%) | 67 (1.8%) | 19 (1.7%) |  |
| ***Risk*** | |  |  |  |  |  | **<0.001** |
|  | Heterosexual | 1,344 (22.7%) | 46 (20.2%) | 244 (26.3%) | 775 (21.2%) | 279 (25.1%) |  |
|  | MSM/IDU | 330 (5.6%) | 18 (7.9%) | 63 (6.8%) | 177 (4.8%) | 72 (6.5%) |  |
|  | MSM | 3,037 (51.3%) | 111 (48.7%) | 431 (46.4%) | 1,937 (53.1%) | 558 (50.3%) |  |
|  | Others | 1,205 (20.4%) | 53 (23.2%) | 190 (20.5%) | 761 (20.8%) | 201 (18.1%) |  |
| ***Region*** | |  |  |  |  |  | 0.166 |
|  | Urban | 4,913 (83.0%) | 191 (83.8%) | 763 (82.2%) | 3,059 (83.8%) | 900 (81.1%) |  |
|  | Rural | 1,003 (17.0%) | 37 (16.2%) | 165 (17.8%) | 591 (16.2%) | 210 (18.9%) |  |
| ***Alcohol Use*** | |  |  |  |  |  | **<0.001** |
|  | No | 4,189 (70.8%) | 155 (68.0%) | 629 (67.8%) | 2,657 (72.8%) | 748 (67.4%) |  |
|  | Yes | 1,727 (29.2%) | 73 (32.0%) | 299 (32.2%) | 993 (27.2%) | 362 (32.6%) |  |
| ***Tobacco Use*** | |  |  |  |  |  | **<0.001** |
|  | No | 4,438 (75.0%) | 164 (71.9%) | 676 (72.8%) | 2,811 (77.0%) | 787 (70.9%) |  |
|  | Yes | 1,478 (25.0%) | 64 (28.1%) | 252 (27.2%) | 839 (23.0%) | 323 (29.1%) |  |
| ***Illicit Drug Use*** | |  |  |  |  |  | **0.007** |
|  | No | 5,444 (92.0%) | 205 (89.9%) | 841 (90.6%) | 3,394 (93.0%) | 1,004 (90.5%) |  |
|  | Yes | 472 (8.0%) | 23 (10.1%) | 87 (9.4%) | 256 (7.0%) | 106 (9.5%) |  |
| ***Comorbidity History*** | |  |  |  |  |  | **<0.001** |
|  | No | 4,303 (72.7%) | 164 (71.9%) | 659 (71.0%) | 2,661 (72.9%) | 819 (73.8%) |  |
|  | Yes | 1,613 (27.2%) | 64 (28.1%) | 269 (29.0%) | 989 (27.1%) | 291 (26.2%) |  |
| ***Baseline VL (copies/mL)*** | |  |  |  |  |  | **<0.001** |
|  | ≤200 | 4,979 (85.9%) | 138 (60.5%) | 623 (67.1%) | 3,404 (96.4%) | 814 (73.3%) |  |
|  | >200 and ≤10,000 | 434 (7.5%) | 25 (11.0%) | 103 (11.1%) | 126 (3.6%) | 180 (16.2%) |  |
|  | >10,000 and ≤100,000 | 241 (4.2%) | 19 (8.3%) | 106 (11.4%) | 0 (0.0%) | 116 (10.5%) |  |
|  | ≥100,000 | 142 (2.4%) | 46 (20.2%) | 96 (10.3%) | 0 (0.0%) | 0 (0.0%) |  |
| ***Last VL (copies/mL)*** | |  |  |  |  |  | **<0.001** |
|  | ≤200 | 5,219 (88.2%) | 135 (59.2%) | 628 (67.7%) | 3,592 (98.4%) | 864 (77.8%) |  |
|  | >200 and ≤10,000 | 279 (4.7%) | 11 (4.8%) | 73 (7.9%) | 58 (1.6%) | 137 (12.3%) |  |
|  | >10,000 and ≤100,000 | 255 (4.3%) | 8 (3.5%) | 138 (14.9%) | 0 (0.0%) | 109 (9.8%) |  |
|  | ≥100,000 | 163 (2.8%) | 74 (32.5%) | 89 (9.6%) | 0 (0.0%) | 0 (0.0%) |  |
| ***Maximum VL (copies/mL)*** | |  |  |  |  |  | **<0.001** |
|  | ≤200 | 3,037 (51.3%) | 0 (0.0%) | 0 (0.0%) | 3,037 (83.2%) | 0 (0.0%) |  |
|  | >200 and ≤10,000 | 925 (15.6%) | 0 (0.0%) | 0 (0.0%) | 613 (16.8%) | 312 (28.1%) |  |
|  | >10,000 and ≤100,000 | 1,009 (17.1%) | 0 (0.0%) | 211 (22.7%) | 0 (0.0%) | 798 (71.9%) |  |
|  | ≥100,000 | 945 (16.0%) | 228 (100.0%) | 717 (77.3%) | 0 (0.0%) | 0 (0.0%) |  |
| ***Mean VL (copies/mL)*** | |  |  |  |  |  | **<0.001** |
|  | ≤200 | 3,037 (51.3%) | 0 (0.0%) | 0 (0.0%) | 3,037 (83.2%) | 0 (0.0%) |  |
|  | >200 and ≤10,000 | 1,884 (31.8%) | 0 (0.0%) | 216 (23.3%) | 613 (16.8%) | 1,055 (95.0%) |  |
|  | >10,000 and ≤100,000 | 784 (13.3%) | 63 (27.6%) | 666 (71.8%) | 0 (0.0%) | 55 (5.0%) |  |
|  | ≥100,000 | 211 (3.6%) | 165 (72.4%) | 46 (5.0%) | 0 (0.0%) | 0 (0.0%) |  |
| ***Percentage of time with Viral Suppression*** | |  |  |  |  |  | **<0.001** |
|  | =0% | 2 (0.0%) | 1 (0.4%) | 1 (0.1%) | 0 (0.0%) | 0 (0.0%) |  |
|  | >0% and ≤25% | 371 (6.3%) | 59 (25.9%) | 164 (17.7%) | 33 (0.9%) | 115 (10.4%) |  |
|  | >25% and ≤50% | 525 (8.9%) | 61 (26.8%) | 233 (25.1%) | 50 (1.4%) | 181 (16.3%) |  |
|  | >50% and ≤75% | 788 (13.3%) | 59 (25.9%) | 257 (27.7%) | 131 (3.6%) | 341 (30.7%) |  |
|  | >75% and <100% | 1,193 (20.2%) | 48 (21.1%) | 273 (29.4%) | 399 (10.9%) | 473 (42.6%) |  |
|  | =100% | 3,037 (51.3%) | 0 (0.0%) | 0 (0.0%) | 3,037 (83.2%) | 0 (0.0%) |  |
| ***Viral Rebound History*** | |  |  |  |  |  | **<0.001** |
|  | No | 4,851 (82.0%) | 114 (50.0%) | 474 (51.1%) | 3,552 (97.3%) | 711 (64.1%) |  |
|  | Yes | 1,065 (18.0%) | 114 (50.0%) | 454 (48.9%) | 98 (2.7%) | 399 (35.9%) |  |
| ***Baseline CD4 Count (copies/μL)*** | |  |  |  |  |  | **<0.001** |
|  | <200 | 1,188 (20.3%) | 82 (36.0%) | 292 (31.5%) | 614 (17.1%) | 200 (18.0%) |  |
|  | ≥200 and <350 | 1,270 (21.7%) | 66 (28.9%) | 213 (23.0%) | 752 (20.9%) | 239 (21.5%) |  |
|  | ≥350 and <500 | 1,288 (22.0%) | 40 (17.5%) | 188 (20.3%) | 811 (22.6%) | 249 (22.4%) |  |
|  | ≥500 | 2,115 (36.1%) | 40 (17.5%) | 234 (25.2%) | 1,419 (39.5%) | 422 (38.0%) |  |
| ***Last CD4 Count (copies/μL)*** | |  |  |  |  |  | **<0.001** |
|  | <200 | 539 (9.2%) | 97 (42.5%) | 255 (27.5%) | 107 (3.0%) | 80 (7.2%) |  |
|  | ≥200 and <350 | 664 (11.3%) | 42 (18.4%) | 192 (20.7%) | 275 (7.6%) | 155 (14.0%) |  |
|  | ≥350 and <500 | 943 (16.1%) | 32 (14.0%) | 162 (17.5%) | 511 (14.2%) | 238 (21.4%) |  |
|  | ≥500 | 3,715 (63.4%) | 57 (25.0%) | 318 (34.3%) | 2,703 (75.2%) | 637 (57.4%) |  |
| ***Maximum CD4 Count (copies/μL)*** | |  |  |  |  |  | **<0.001** |
|  | <200 | 119 (2.0%) | 15 (6.6%) | 49 (5.3%) | 43 (1.2%) | 12 (1.1%) |  |
|  | ≥200 and <350 | 353 (6.0%) | 36 (15.8%) | 117 (12.6%) | 132 (3.7%) | 68 (6.1%) |  |
|  | ≥350 and <500 | 594 (10.1%) | 46 (20.2%) | 155 (16.7%) | 278 (7.7%) | 115 (10.4%) |  |
|  | ≥500 | 4,795 (81.8%) | 131 (57.5%) | 606 (65.4%) | 3,143 (87.4%) | 915 (82.4%) |  |
| ***Mean CD4 Count (copies/μL)*** | |  |  |  |  |  | **<0.001** |
|  | <200 | 460 (7.8%) | 81 (35.5%) | 195 (21.0%) | 118 (3.3%) | 66 (5.9%) |  |
|  | ≥200 and <350 | 867 (14.8%) | 67 (29.4%) | 236 (25.5%) | 387 (10.8%) | 177 (15.9%) |  |
|  | ≥350 and <500 | 1,159 (19.8%) | 43 (18.9%) | 209 (22.5%) | 656 (18.2%) | 251 (22.6%) |  |
|  | ≥500 | 3,375 (57.6%) | 37 (16.2%) | 287 (31.0%) | 2,435 (67.7%) | 616 (55.5%) |  |
| ***Minimum CD4 Count (copies/μL)*** | |  |  |  |  |  | **<0.001** |
|  | <200 | 2,025 (34.6%) | 195 (85.5%) | 601 (64.8%) | 845 (23.5%) | 384 (34.6%) |  |
|  | ≥200 and <350 | 1,427 (24.3%) | 19 (8.3%) | 190 (20.5%) | 897 (24.9%) | 321 (28.9%) |  |
|  | ≥350 and <500 | 1,229 (21.0%) | 6 (2.6%) | 95 (10.2%) | 880 (24.5%) | 248 (22.3%) |  |
|  | ≥500 | 1,180 (20.1%) | 8 (3.5%) | 41 (4.4%) | 974 (27.1%) | 157 (14.1%) |  |
| ***Percentage of Time with Low CD4 Count*** | |  |  |  |  |  | **<0.001** |
|  | =0% | 3,885 (66.3%) | 37 (16.2%) | 345 (37.2%) | 2,766 (76.9%) | 737 (66.4%) |  |
|  | >0% and ≤25% | 1,381 (23.6%) | 92 (40.4%) | 341 (36.8%) | 676 (18.8%) | 272 (24.5%) |  |
|  | >25% and ≤50% | 293 (5.0%) | 56 (24.6%) | 113 (12.2%) | 78 (2.2%) | 46 (4.1%) |  |
|  | >50% and ≤75% | 124 (2.1%) | 19 (8.3%) | 58 (6.3%) | 18 (0.5%) | 29 (2.6%) |  |
|  | >75% and <100% | 59 (1.0%) | 9 (3.9%) | 21 (2.3%) | 15 (0.4%) | 14 (1.3%) |  |
| ***VL 2nd percentile (copies/mL)*** | |  |  |  |  |  | **<0.001** |
|  | ≤200 | 5,833 (98.6%) | 205 (89.9%) | 898 (96.8%) | 3,645 (99.9%) | 1,085 (97.7%) |  |
|  | >200 and ≤10,000 | 76 (1.3%) | 16 (7.0%) | 30 (3.2%) | 5 (0.1%) | 25 (2.3%) |  |
|  | >10,000 and ≤100,000 | 7 (0.1%) | 7 (3.1%) | 0 (0.0%) | 0 (0.0%) | 0 (0.0%) |  |
|  | ≥100,000 | 0 (0.0%) | 0 (0.0%) | 0 (0.0%) | 0 (0.0%) | 0 (0.0%) |  |
| ***VL 5th percentile (copies/mL)*** | |  |  |  |  |  | **<0.001** |
|  | ≤200 | 5,829 (98.5%) | 205 (89.9%) | 895 (96.4%) | 3,645 (99.9%) | 1,084 (97.7%) |  |
|  | >200 and ≤10,000 | 73 (1.2%) | 14 (6.1%) | 28 (3.0%) | 5 (0.1%) | 26 (2.3%) |  |
|  | >10,000 and ≤100,000 | 13 (0.2%) | 8 (3.5%) | 5 (0.5%) | 0 (0.0%) | 0 (0.0%) |  |
|  | ≥100,000 | 1 (0.0%) | 1 (0.4%) | 0 (0.0%) | 0 (0.0%) | 0 (0.0%) |  |
| ***VL 95th percentile (copies/mL)*** | |  |  |  |  |  | **<0.001** |
|  | ≤200 | 3,324 (56.2%) | 6 (2.6%) | 32 (3.4%) | 3,203 (87.8%) | 83 (7.5%) |  |
|  | >200 and ≤10,000 | 1,050 (17.7%) | 7 (3.1%) | 68 (7.3%) | 447 (12.2%) | 528 (47.6%) |  |
|  | >10,000 and ≤100,000 | 906 (15.3%) | 14 (6.1%) | 393 (42.3%) | 0 (0.0%) | 499 (45.0%) |  |
|  | ≥100,000 | 636 (10.8%) | 201 (88.2%) | 435 (46.9%) | 0 (0.0%) | 0 (0.0%) |  |
| ***VL 98th percentile (copies/mL)*** | |  |  |  |  |  | **<0.001** |
|  | ≤200 | 3,039 (51.4%) | 0 (0.0%) | 1 (0.1%) | 3,038 (83.2%) | 0 (0.0%) |  |
|  | >200 and ≤10,000 | 1,035 (17.5%) | 0 (0.0%) | 3 (0.3%) | 612 (16.8%) | 420 (37.8%) |  |
|  | >10,000 and ≤100,000 | 997 (16.9%) | 1 (0.4%) | 306 (33.0%) | 0 (0.0%) | 690 (62.2%) |  |
|  | ≥100,000 | 845 (14.3%) | 227 (99.6%) | 618 (66.6%) | 0 (0.0%) | 0 (0.0%) |  |
| ^1^ P-values were calculated using Pearson's Chi-squared test. | | |  |  |  |  |  |
| ^2^ Unable to calculate part of the P-values because the counts of some groups are too small. | | | | | | | |

Supplementary Table 3: Distribution for VL characteristics and percentiles, demographics, comorbidities, social behaviors, and historical CD4 count for overall and four clusters from K-means on the same FPC scores

| **Characteristics** | | **Overall**, **N** = 5,916 | Cluster 1 | Cluster 2 | Cluster 3 | Cluster 4 | **P value^12^** |
| --- | --- | --- | --- | --- | --- | --- | --- |
|  |  |  | n=894 (15.1%) | n=454 (7.7%) | n=4,378 (74.0%) | n=190 (3.2%) |  |
| ***Age Group (Years)*** | |  |  |  |  |  | **<0.001** |
|  | ≥18 and <30 | 2,362 (39.9%) | 433 (48.4%) | 216 (47.6%) | 1,612 (36.8%) | 101 (53.2%) |  |
|  | ≥30 and <40 | 1,367 (23.1%) | 216 (24.2%) | 120 (26.4%) | 990 (22.6%) | 41 (21.6%) |  |
|  | ≥40 and <50 | 1,327 (22.4%) | 167 (18.7%) | 91 (20.0%) | 1,036 (23.7%) | 33 (17.4%) |  |
|  | ≥50 | 860 (14.5%) | 78 (8.8%) | 27 (6.0%) | 740 (16.9%) | 15 (7.9%) |  |
| ***Sex*** | |  |  |  |  |  | 0.089 |
|  | Male | 4,380 (74.0%) | 646 (72.3%) | 329 (72.5%) | 3,275 (74.8%) | 130 (68.4%) |  |
|  | Female | 1,536 (26.0%) | 248 (27.7%) | 125 (27.5%) | 1,103 (25.2%) | 60 (31.6%) |  |
| ***Race*** | |  |  |  |  |  |  |
|  | White | 1,316 (22.2%) | 143 (16.0%) | 71 (15.6%) | 1,070 (24.4%) | 32 (16.8%) |  |
|  | Black | 4,269 (72.2%) | 705 (78.9%) | 361 (79.5%) | 3,050 (69.7%) | 153 (80.5%) |  |
|  | Hispanic | 225 (3.8%) | 27 (3.0%) | 15 (3.3%) | 181 (4.1%) | 2 (1.1%) |  |
|  | Others | 106 (1.8%) | 19 (2.1%) | 7 (1.5%) | 77 (1.8%) | 3 (1.6%) |  |
| ***Risk*** | |  |  |  |  |  | **0.006** |
|  | Heterosexual | 1,344 (22.7%) | 220 (24.6%) | 119 (26.2%) | 959 (21.9%) | 46 (24.2%) |  |
|  | MSM/IDU | 330 (5.6%) | 62 (6.9%) | 35 (7.7%) | 218 (5.0%) | 15 (7.9%) |  |
|  | MSM | 3,037 (51.3%) | 439 (49.1%) | 219 (48.2%) | 2,293 (52.4%) | 86 (45.3%) |  |
|  | Others | 1,205 (20.4%) | 173 (19.4%) | 81 (17.8%) | 908 (20.7%) | 43 (22.6%) |  |
| ***Region*** | |  |  |  |  |  | 0.719 |
|  | Urban | 4,913 (83.0%) | 736 (82.3%) | 372 (81.9%) | 3,650 (83.4%) | 155 (81.6%) |  |
|  | Rural | 1,003 (17.0%) | 158 (17.7%) | 82 (18.1%) | 728 (16.6%) | 35 (18.4%) |  |
| ***Alcohol Use*** | |  |  |  |  |  | **<0.001** |
|  | No | 4,189 (70.8%) | 591 (66.1%) | 314 (69.2%) | 3,168 (72.4%) | 116 (61.1%) |  |
|  | Yes | 1,727 (29.2%) | 303 (33.9%) | 140 (30.8%) | 1,210 (27.6%) | 74 (38.9%) |  |
| ***Tobacco Use*** | |  |  |  |  |  | **<0.001** |
|  | No | 4,438 (75.0%) | 627 (70.1%) | 341 (75.1%) | 3,345 (76.4%) | 125 (65.8%) |  |
|  | Yes | 1,478 (25.0%) | 267 (29.9%) | 113 (24.9%) | 1,033 (23.6%) | 65 (34.2%) |  |
| ***Illicit Drug Use*** | |  |  |  |  |  | **<0.001** |
|  | No | 5,444 (92.0%) | 805 (90.0%) | 407 (89.6%) | 4,068 (92.9%) | 164 (86.3%) |  |
|  | Yes | 472 (8.0%) | 89 (10.0%) | 47 (10.4%) | 310 (7.1%) | 26 (13.7%) |  |
| ***Comorbidity History*** | |  |  |  |  |  | 0.732 |
|  | No | 4,303 (72.7%) | 659 (73.7%) | 340 (74.9%) | 3,171 (72.4%) | 133 (70.0%) |  |
|  | Yes | 1,613 (27.2%) | 235 (26.3%) | 114 (25.1%) | 1,207 (27.6%) | 57 (30.0%) |  |
| ***Baseline VL (copies/mL)*** | |  |  |  |  |  | **<0.001** |
|  | ≤200 | 4,979 (85.9%) | 600 (67.1%) | 288 (63.4%) | 3,994 (93.8%) | 97 (51.1%) |  |
|  | >200 and ≤10,000 | 434 (7.5%) | 149 (16.7%) | 72 (15.9%) | 185 (4.3%) | 28 (14.7%) |  |
|  | >10,000 and ≤100,000 | 241 (4.2%) | 91 (10.2%) | 59 (13.0%) | 57 (1.3%) | 34 (17.9%) |  |
|  | ≥100,000 | 142 (2.4%) | 54 (6.0%) | 35 (7.7%) | 22 (0.5%) | 31 (16.3%) |  |
| ***Last VL (copies/mL)*** | |  |  |  |  |  | **<0.001** |
|  | ≤200 | 5,219 (88.2%) | 687 (76.8%) | 189 (41.6%) | 4,301 (98.2%) | 42 (22.1%) |  |
|  | >200 and ≤10,000 | 279 (4.7%) | 124 (13.9%) | 74 (16.3%) | 64 (1.5%) | 17 (8.9%) |  |
|  | >10,000 and ≤100,000 | 255 (4.3%) | 69 (7.7%) | 130 (28.6%) | 11 (0.3%) | 45 (23.7%) |  |
|  | ≥100,000 | 163 (2.8%) | 14 (1.6%) | 61 (13.4%) | 2 (0.0%) | 86 (45.3%) |  |
| ***Maximum VL (copies/mL)*** | |  |  |  |  |  | **<0.001** |
|  | ≤200 | 3,037 (51.3%) | 0 (0.0%) | 0 (0.0%) | 3,037 (69.4%) | 0 (0.0%) |  |
|  | >200 and ≤10,000 | 925 (15.6%) | 149 (16.7%) | 10 (2.2%) | 766 (17.5%) | 0 (0.0%) |  |
|  | >10,000 and ≤100,000 | 1,009 (17.1%) | 414 (46.3%) | 166 (36.6%) | 413 (9.4%) | 16 (8.4%) |  |
|  | ≥100,000 | 945 (16.0%) | 331 (37.0%) | 278 (61.2%) | 162 (3.7%) | 174 (91.6%) |  |
| ***Mean VL (copies/mL)*** | |  |  |  |  |  | **<0.001** |
|  | ≤200 | 3,037 (51.3%) | 0 (0.0%) | 0 (0.0%) | 3,037 (69.4%) | 0 (0.0%) |  |
|  | >200 and ≤10,000 | 1,884 (31.8%) | 541 (60.5%) | 99 (21.8%) | 1,244 (28.4%) | 0 (0.0%) |  |
|  | >10,000 and ≤100,000 | 784 (13.3%) | 317 (35.5%) | 301 (66.3%) | 85 (1.9%) | 81 (42.6%) |  |
|  | ≥100,000 | 211 (3.6%) | 36 (4.0%) | 54 (11.9%) | 12 (0.3%) | 109 (57.4%) |  |
| ***VL 2nd percentile (copies/mL)*** | |  |  |  |  |  | / |
|  | ≤200 | 5,833 (98.6%) | 882 (98.7%) | 426 (93.8%) | 4,375 (99.9%) | 150 (78.9%) |  |
|  | >200 and ≤10,000 | 76 (1.3%) | 12 (1.3%) | 28 (6.2%) | 3 (0.1%) | 33 (17.4%) |  |
|  | >10,000 and ≤100,000 | 7 (0.1%) | 0 (0.0%) | 0 (0.0%) | 0 (0.0%) | 7 (3.7%) |  |
|  | ≥100,000 | 0 (0.0%) | 0 (0.0%) | 0 (0.0%) | 0 (0.0%) | 0 (0.0%) |  |
| ***VL 5th percentile (copies/mL)*** | |  |  |  |  |  | / |
|  | ≤200 | 5,829 (98.5%) | 882 (98.7%) | 425 (93.6%) | 4,375 (99.9%) | 147 (77.4%) |  |
|  | >200 and ≤10,000 | 73 (1.2%) | 12 (1.3%) | 27 (5.9%) | 2 (0.0%) | 32 (16.8%) |  |
|  | >10,000 and ≤100,000 | 13 (0.2%) | 0 (0.0%) | 2 (0.4%) | 1 (0.0%) | 10 (5.3%) |  |
|  | ≥100,000 | 1 (0.0%) | 0 (0.0%) | 0 (0.0%) | 0 (0.0%) | 1 (0.5%) |  |
| ***VL 95th percentile (copies/mL)*** | |  |  |  |  |  | **<0.001** |
|  | ≤200 | 3,324 (56.2%) | 1 (0.1%) | 0 (0.0%) | 3,323 (75.9%) | 0 (0.0%) |  |
|  | >200 and ≤10,000 | 1,050 (17.7%) | 221 (24.7%) | 16 (3.5%) | 813 (18.6%) | 0 (0.0%) |  |
|  | >10,000 and ≤100,000 | 906 (15.3%) | 467 (52.2%) | 206 (45.4%) | 212 (4.8%) | 21 (11.1%) |  |
|  | ≥100,000 | 636 (10.8%) | 205 (22.9%) | 232 (51.1%) | 30 (0.7%) | 169 (88.9%) |  |
| ***VL 98th percentile (copies/mL)*** | |  |  |  |  |  | **<0.001** |
|  | ≤200 | 3,039 (51.4%) | 0 (0.0%) | 0 (0.0%) | 3,039 (69.4%) | 0 (0.0%) |  |
|  | >200 and ≤10,000 | 1,035 (17.5%) | 165 (18.5%) | 11 (2.4%) | 859 (19.6%) | 0 (0.0%) |  |
|  | >10,000 and ≤100,000 | 997 (16.9%) | 434 (48.5%) | 174 (38.3%) | 371 (8.5%) | 18 (9.5%) |  |
|  | ≥100,000 | 845 (14.3%) | 295 (33.0%) | 269 (59.3%) | 109 (2.5%) | 172 (90.5%) |  |
| ***Baseline CD4 Count (copies/μL)*** | |  |  |  |  |  | **<0.001** |
|  | <200 | 1,188 (20.3%) | 219 (24.5%) | 121 (26.7%) | 793 (18.3%) | 55 (28.9%) |  |
|  | ≥200 and <350 | 1,270 (21.7%) | 183 (20.5%) | 97 (21.4%) | 942 (21.8%) | 48 (25.3%) |  |
|  | ≥350 and <500 | 1,288 (22.0%) | 185 (20.7%) | 107 (23.6%) | 957 (22.1%) | 39 (20.5%) |  |
|  | ≥500 | 2,115 (36.1%) | 306 (34.3%) | 128 (28.3%) | 1,633 (37.8%) | 48 (25.3%) |  |
| ***Last CD4 Count (copies/μL)*** | |  |  |  |  |  | **<0.001** |
|  | <200 | 539 (9.2%) | 108 (12.1%) | 165 (36.4%) | 142 (3.3%) | 124 (65.3%) |  |
|  | ≥200 and <350 | 664 (11.3%) | 170 (19.0%) | 118 (26.0%) | 341 (7.9%) | 35 (18.4%) |  |
|  | ≥350 and <500 | 943 (16.1%) | 190 (21.3%) | 82 (18.1%) | 655 (15.1%) | 16 (8.4%) |  |
|  | ≥500 | 3,715 (63.4%) | 425 (47.6%) | 88 (19.4%) | 3,187 (73.7%) | 15 (7.9%) |  |
| ***Maximum CD4 Count (copies/μL)*** | |  |  |  |  |  | **<0.001** |
|  | <200 | 119 (2.0%) | 23 (2.6%) | 27 (6.0%) | 45 (1.0%) | 24 (12.6%) |  |
|  | ≥200 and <350 | 353 (6.0%) | 79 (8.8%) | 71 (15.7%) | 161 (3.7%) | 42 (22.1%) |  |
|  | ≥350 and <500 | 594 (10.1%) | 128 (14.3%) | 77 (17.0%) | 348 (8.0%) | 41 (21.6%) |  |
|  | ≥500 | 4,795 (81.8%) | 663 (74.2%) | 278 (61.4%) | 3,771 (87.2%) | 83 (43.7%) |  |
| ***Mean CD4 Count (copies/μL)*** | |  |  |  |  |  | **<0.001** |
|  | <200 | 460 (7.8%) | 102 (11.4%) | 113 (24.9%) | 151 (3.5%) | 94 (49.5%) |  |
|  | ≥200 and <350 | 867 (14.8%) | 201 (22.5%) | 121 (26.7%) | 496 (11.5%) | 49 (25.8%) |  |
|  | ≥350 and <500 | 1,159 (19.8%) | 214 (24.0%) | 99 (21.9%) | 823 (19.0%) | 23 (12.1%) |  |
|  | ≥500 | 3,375 (57.6%) | 376 (42.1%) | 120 (26.5%) | 2,855 (66.0%) | 24 (12.6%) |  |
| ***Minimum CD4 Count (copies/μL)*** | |  |  |  |  |  | **<0.001** |
|  | <200 | 2,025 (34.6%) | 432 (48.4%) | 298 (65.8%) | 1,133 (26.2%) | 162 (85.3%) |  |
|  | ≥200 and <350 | 1,427 (24.3%) | 213 (23.9%) | 82 (18.1%) | 1,115 (25.8%) | 17 (8.9%) |  |
|  | ≥350 and <500 | 1,229 (21.0%) | 156 (17.5%) | 50 (11.0%) | 1,014 (23.4%) | 9 (4.7%) |  |
|  | ≥500 | 1,180 (20.1%) | 92 (10.3%) | 23 (5.1%) | 1,063 (24.6%) | 2 (1.1%) |  |
| *1* n (%) | | | | | | | |
| *2* Pearson’s Chi-squared test | | | | | | | |
